# Supplementary material for: CalcAMP: A New Machine Learning Model for the Accurate Prediction of Antimicrobial Activity of Peptides
Source: Antibiotics (Basel). 2023 Apr 7;12(4):725. doi: 10.3390/antibiotics12040725 (PMC10135148; doi:10.3390/antibiotics12040725)
Supplement: Supplementary file 1 [file antibiotics-12-00725-s001.zip › antibiotics-2277614-supplementary.pdf]

antibiotics

Article

Supplemental information

CalcAMP: A new machine learning model for the accurate calculation of antimicrobial activity of peptides

Colin Bournez[1], Martijn Riool[2,†], Leonie de Boer[2], Robert A. Cordfunke[3], Leonie de Best[4], Remko van Leeuwen[4], Jan Wouter Drijfhout[3], Sebastian A.J. Zaat[2], Gerard J.P. van Westen[1]*

[1] Division of Medicinal Chemistry, Drug Discovery and Safety, Leiden Academic Centre for Drug Research, Leiden University, P.O. Box 9502, 2300 RA, Leiden, The Netherlands
[2] Department of Medical Microbiology and Infection Prevention, Amsterdam institute for Infection and Immunity, Amsterdam UMC, University of Amsterdam, 1105 AZ Amsterdam, The Netherlands
[†] Current address: Laboratory for Experimental Trauma Surgery, Department of Trauma Surgery, University Medical Center Regensburg, Am Biopark 9, 93053, Regensburg, Germany
[3] Department Immunology, Leiden University Medical Center, 2300 RC Leiden, The Netherlands
[4] Madam Therapeutics B.V., Pivot Park Life Sciences Community, Kloosterstraat 9, 5349AB Oss, The Netherlands
* Correspondence: gerard@lacdr.leidenuniv.nl

Citation: Lastname, F.; Lastname, F.; Lastname, F. Title. *Antibiotics* **2023**, *12*, 725. https://doi.org/10.3390/antibiotics12040725

Academic Editor: Fernando Albericio

Received: date
Accepted: date
Published: 7 April 2023

Copyright: © 2023 by the author. Licensee MDPI, Basel, Switzerland. This article is an open access article distributed under the terms and conditions of the Creative Commons Attribution (CC BY) license (https://creativecommons.org/licenses/by/4.0/).

- **Dataset exploration**

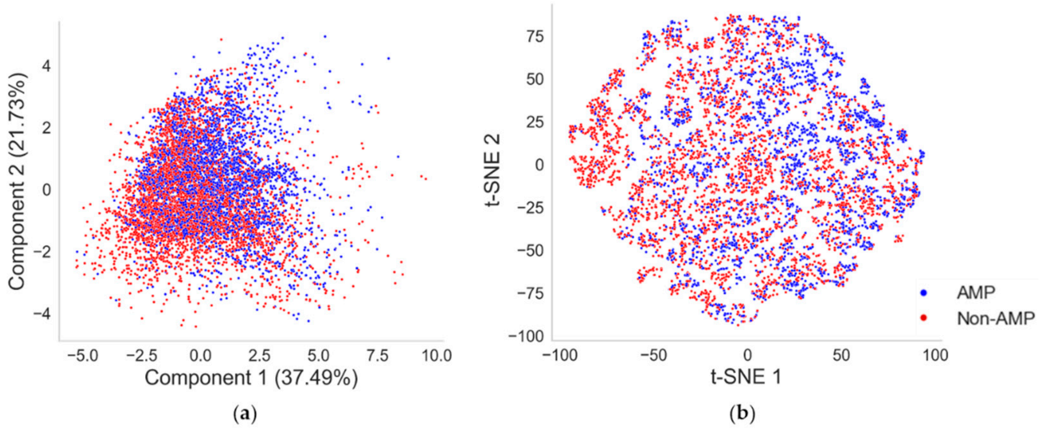

Figure S1. PCA (**a**) and t-SNE (**b**) projections of physicochemical descriptors between AMPs and Non-AMPs.

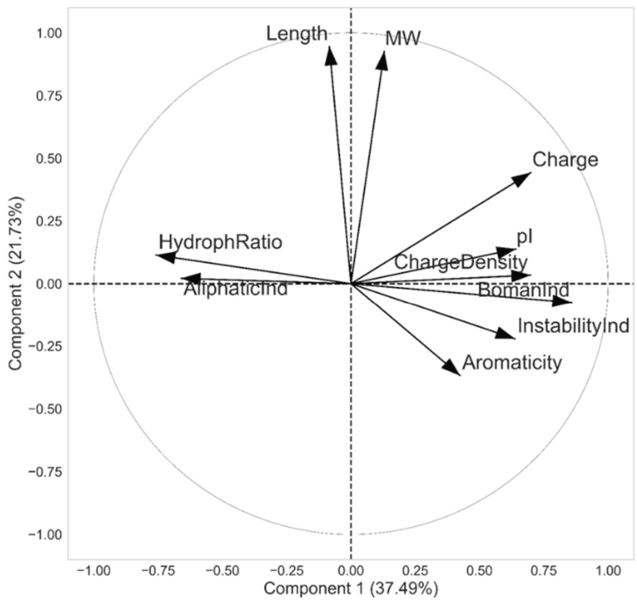

Correlation circle associated to PCA shown in Figure S1.

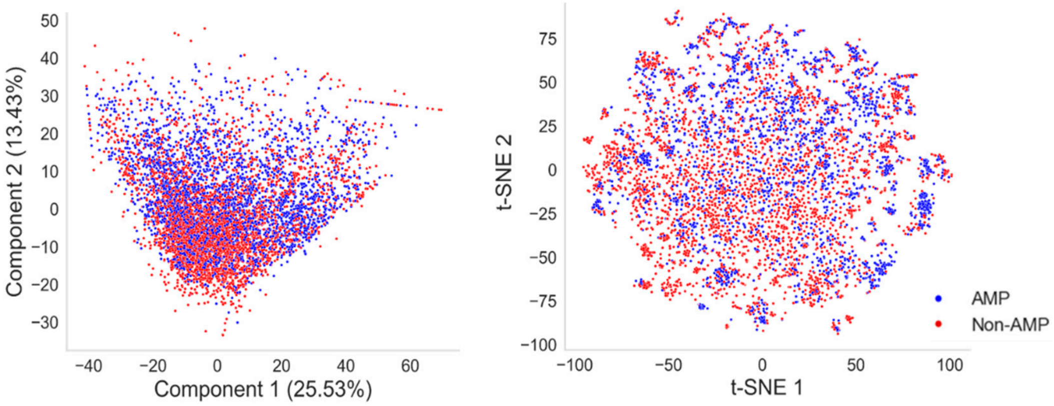

(**a**) (**b**)

Figure S2. PCA (a) and t-SNE (b) projections of amino acid composition between AMPs and Non-AMPs.

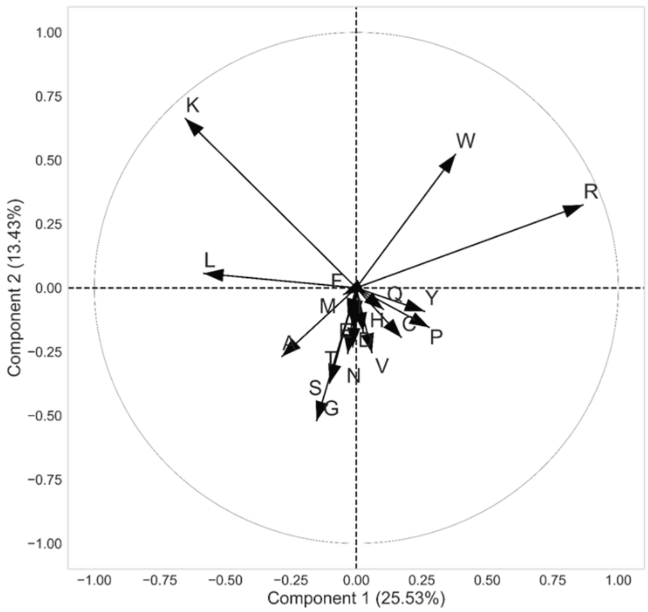

Correlation circle associated to PCA shown in Figure S2.

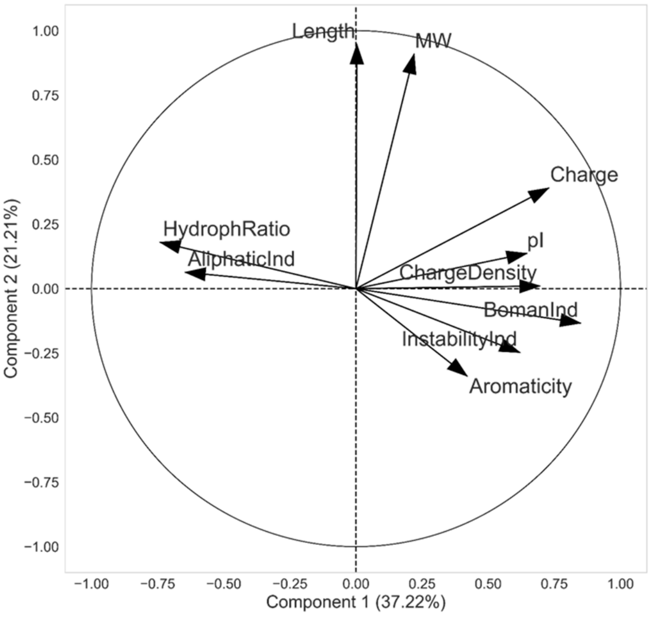

Figure S3. Correlation circle associated to PCA shown in Figure 5 in the main text.

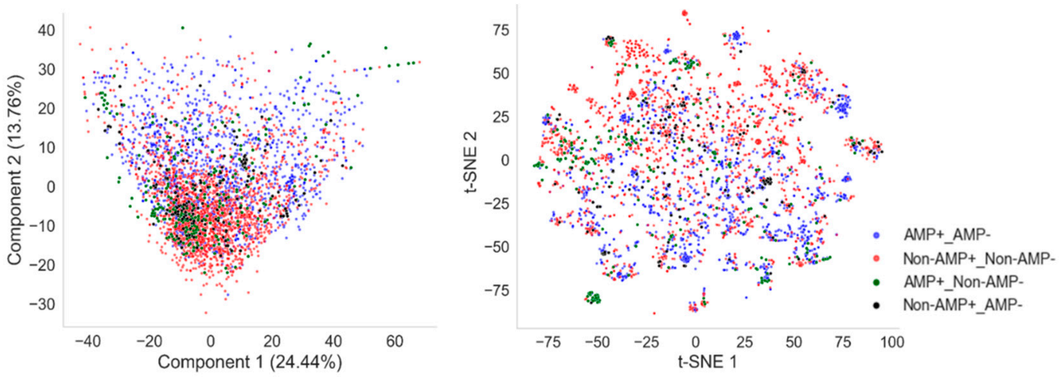

(**a**) (**b**)

Figure S4. PCA (**a**) and t-SNE (**b**) projections of amino acid composition between common peptides of Gram+ and Gram- categories. In blue, peptides are labelled as AMP in both categories, in red both are Non-AMP, in green peptides are AMP for Gram+ and Non-AMP for Gram- and in black the opposite.

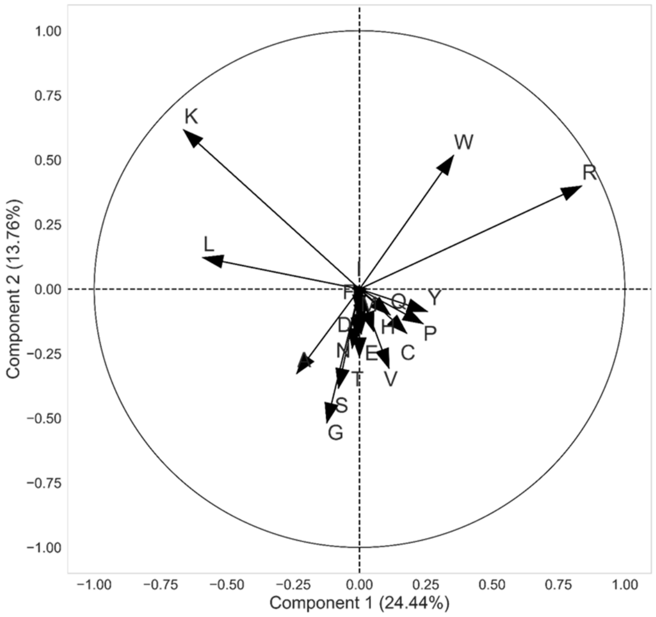

Correlation circle associated to PCA shown in Figure S4.

- **ML model comparison**

Table S1. Comparison of ML classifiers tested for Gram+ AMP prediction (10 Times 10-Fold Cross-Validation. The values in brackets represent the standard deviation obtained via 10-fold cross validation.

Model	Accuracy	Sensitivity	Specificity	AUC-ROC	MCC
LGBMClassifier	0.80 (0.02)	0.81 (0.02)	0.78 (0.02)	0.88 (0.01)	0.59 (0.03)
RandomForestClassifier	0.80 (0.02)	0.80 (0.03)	0.80 (0.03)	0.88 (0.01)	0.60 (0.04)
ExtraTreesClassifier	0.80 (0.02)	0.80 (0.03)	0.80 (0.03)	0.88 (0.02)	0.60 (0.04)
XGBClassifier	0.80 (0.02)	0.81 (0.02)	0.78 (0.02)	0.88 (0.01)	0.59 (0.03)
CatBoostClassifier	0.80 (0.01)	0.83 (0.02)	0.78 (0.02)	0.88 (0.01)	0.61 (0.02)
GradientBoostingClassifier	0.79 (0.01)	0.81 (0.02)	0.76 (0.02)	0.86 (0.01)	0.57 (0.02)
AdaBoostClassifier	0.74 (0.02)	0.76 (0.02)	0.71 (0.02)	0.81 (0.01)	0.47 (0.03)
KNeighborsClassifier	0.73 (0.02)	0.79 (0.03)	0.67 (0.04)	0.8 (0.02)	0.47 (0.04)
LogisticRegression	0.73 (0.02)	0.76 (0.02)	0.70 (0.03)	0.79 (0.02)	0.46 (0.04)
DecisionTreeClassifier	0.72 (0.02)	0.74 (0.03)	0.70 (0.03)	0.72 (0.02)	0.44 (0.04)
RidgeClassifier	0.72 (0.02)	0.77 (0.02)	0.66 (0.04)	-	0.44 (0.04)
LinearDiscriminantAnalysis	0.72 (0.02)	0.78 (0.03)	0.67 (0.03)	0.78 (0.02)	0.45 (0.04)
Multi-layer Perceptron	0.68 (0.06)	0.76 (0.24)	0.60 (0.25)	0.80 (0.02)	0.41 (0.08)
GaussianNB	0.67 (0.02)	0.79 (0.03)	0.55 (0.03)	0.7 (0.02)	0.35 (0.04)
SGDClassifier	0.65 (0.08)	0.64 (0.29)	0.66 (0.34)	0.0 (0.0)	0.35 (0.13)
QuadraticDiscriminantAnalysis	0.51 (0.03)	0.49 (0.22)	0.54 (0.18)	0.51 (0.03)	0.03 (0.05)
DummyClassifier	0.51 (0.0)	1.0 (0.0)	0.0 (0.0)	0.50 (0.0)	0.0 (0.0)

Table S2. Comparison of ML classifiers tested for Gram- AMP prediction (10 Times 10-Fold Cross-Validation. The values in brackets represent the standard deviation obtained via 10-fold cross validation.

Model	Accuracy	Sensitivity	Specificity	AUC-ROC	MCC
CatBoostClassifier	0.80 (0.03)	0.79 (0.05)	0.81 (0.03)	0.87 (0.02)	0.60 (0.06)
LGBMClassifier	0.80 (0.02)	0.79 (0.04)	0.81 (0.04)	0.87 (0.02)	0.60 (0.05)
RandomForestClassifier	0.80 (0.02)	0.78 (0.04)	0.81 (0.03)	0.88 (0.02)	0.60 (0.04)
XGBClassifier	0.80 (0.02)	0.79 (0.04)	0.81 (0.04)	0.88 (0.02)	0.60 (0.04)
ExtraTreesClassifier	0.79 (0.02)	0.78 (0.04)	0.81 (0.03)	0.87 (0.02)	0.59 (0.04)
GradientBoostingClassifier	0.78 (0.02)	0.78 (0.05)	0.78 (0.03)	0.85 (0.02)	0.56 (0.05)
Multi-layer Perceptron	0.74 (0.03)	0.80 (0.10)	0.68 (0.11)	0.82 (0.02)	0.49 (0.04)
KNeighborsClassifier	0.74 (0.02)	0.77 (0.03)	0.71 (0.03)	0.81 (0.02)	0.48 (0.03)
LogisticRegression	0.74 (0.02)	0.75 (0.04)	0.73 (0.03)	0.82 (0.02)	0.49 (0.04)
AdaBoostClassifier	0.74 (0.02)	0.74 (0.03)	0.74 (0.02)	0.81 (0.02)	0.49 (0.04)
RidgeClassifier	0.73 (0.03)	0.76 (0.03)	0.70 (0.04)	-	0.46 (0.05)
LinearDiscriminantAnalysis	0.72 (0.02)	0.76 (0.03)	0.69 (0.04)	0.79 (0.03)	0.45 (0.04)
DecisionTreeClassifier	0.71 (0.02)	0.72 (0.03)	0.69 (0.04)	0.71 (0.02)	0.41 (0.04)
GaussianNB	0.67 (0.03)	0.76 (0.03)	0.58 (0.04)	0.71 (0.03)	0.35 (0.05)
SGDClassifier	0.66 (0.09)	0.62 (0.32)	0.70 (0.28)	0.0 (0.0)	0.36 (0.17)
QuadraticDiscriminantAnalysis	0.55 (0.04)	0.71 (0.03)	0.40 (0.09)	0.56 (0.04)	0.12 (0.09)
DummyClassifier	0.51 (0.0)	0.0 (0.0)	1.0 (0.0)	0.50 (0.0)	0.0 (0.0)

- **CalcAMP prediction results**

Table S3. Results of ML classifiers created for Gram+ AMP prediction. The values in brackets represent the standard deviation obtained via 10-fold cross validation.

Model	Accuracy	Sensitivity	Specificity	AUC-ROC	MCC
RF_tuned	0.81 (0.01)	0.81 (0.03)	0.82 (0.01)	0.89 (0.02)	0.63 (0.02)
RF_tuned_sel	0.82 (0.01)	0.81 (0.03)	0.82 (0.03)	0.89 (0.01)	0.63 (0.03)
ET_tuned	0.80 (0.02)	0.80 (0.02)	0.81 (0.02)	0.88 (0.02)	0.61 (0.04)
ET_tuned_sel	0.81 (0.01)	0.81 (0.03)	0.82 (0.01)	0.89 (0.01)	0.63 (0.03)
XGBoost _tuned	0.80 (0.02)	0.81 (0.02)	0.80 (0.02)	0.88 (0.01)	0.61 (0.04)
XGBoost_tuned_sel	0.81 (0.01)	0.81 (0.02)	0.81 (0.02)	0.89 (0.02)	0.62 (0.03)
LightGBM_tuned	0.81 (0.02)	0.81 (0.03)	0.81 (0.03)	0.88 (0.02)	0.62 (0.04)
LightGBM_tuned_sel	0.81 (0.02)	0.81 (0.03)	0.81 (0.03)	0.89 (0.02)	0.63 (0.04)
CatBoost_tuned	0.80 (0.01)	0.81 (0.03)	0.79 (0.03)	0.87 (0.02)	0.60 (0.02)
CatBoost_tuned_sel	0.81 (0.01)	0.82 (0.02)	0.80 (0.03)	0.88 (0.01)	0.62 (0.02)

Table S4. Results of ML classifiers created for Gram- AMP prediction. The values in brackets represent the standard deviation obtained via 10-fold cross validation.

Model	Accuracy	Sensitivity	Specificity	AUC-ROC	MCC
RF_tuned	0.81 (0.02)	0.81 (0.03)	0.82 (0.01)	0.89 (0.01)	0.63 (0.04)
RF_tuned_sel	0.81 (0.02)	0.80 (0.02)	0.81 (0.02)	0.89 (0.02)	0.61 (0.03)
ET_tuned	0.81 (0.01)	0.82 (0.02)	0.80 (0.02)	0.89 (0.01)	0.62 (0.02)
ET_tuned_sel	0.81 (0.01)	0.80 (0.02)	0.81 (0.02)	0.89 (0.01)	0.61 (0.03)
XGBoost _tuned	0.81 (0.01)	0.82 (0.02)	0.81 (0.02)	0.89 (0.01)	0.63 (0.03)
XGBoost_tuned_sel	0.82 (0.01)	0.82 (0.03)	0.82 (0.02)	0.90 (0.02)	0.64 (0.03)
LightGBM_tuned	0.81 (0.01)	0.81 (0.03)	0.82 (0.03)	0.89 (0.01)	0.63 (0.03)
LightGBM_tuned_sel	0.80 (0.02)	0.80 (0.03)	0.80 (0.03)	0.88 (0.01)	0.60 (0.04)
CatBoost_tuned	0.80 (0.01)	0.80 (0.02)	0.79 (0.02)	0.88 (0.01)	0.60 (0.03)
CatBoost_tuned_sel	0.81 (0.02)	0.81 (0.02)	0.81 (0.03)	0.89 (0.02)	0.62 (0.04)

- **Comparison with other datasets**

CalcAMP was compared to four existing tools freely available:

- iAMPpred (http://cabgrid.res.in:8080/amppred/server.php)
- DBAASP (https://dbaasp.org/tools?page=general-prediction)
- RF-AmPEP30 and Deep-AmPEP30 (https://cbbio.online/AxPEP/)
- AMP Scanner Vr.2 (https://www.dveltri.com/ascan/v2/ascan.html)

Comparison with AmPEP dataset

The AmPEP benchmark dataset is composed of 94 AMPs and 94 Non-AMPs (sequences with 5-30 AA in length) and was downloaded on their website: https://cbbio.online/AxPEP/?action=dataset. No modification were performed on this dataset and it were used as it was presented. However, it is important to note that this dataset presents several peptides in common with the training dataset of our models. The common peptides are the same for both the Gram+ and Gram- dataset and are shown in figure S5 with a confusion matrix representing how they are labelled in the respective datasets. In particularly, on their 94 peptides classified as AMP: 17 are common to CalcAMP training dataset whom 6 of them are labelled as Non-AMP, contrarily to AmPEP dataset. On their negatives peptides, only 3 are common whom 1 is not classified similarly.

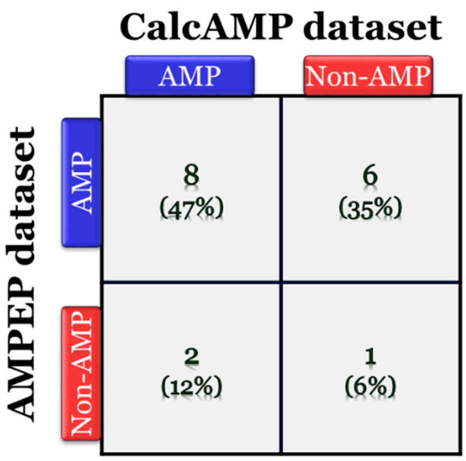

Figure S5. Matrix of labels for the common peptides between training dataset of CalcAMP+ and CalcAMP- models and AmPEP external benchmark dataset.

Table S5. Comparison of different AMP prediction classifiers using AmPEP benchmark dataset.

Model	Accuracy	Sensitivity	Specificity	AUC-ROC	MCC
Deep-AmPEP30	0.84	0.89	0.78	0.93	0.67
RF-AmPEP30	**0.88**	**0.98**	0.79	**0.95**	**0.78**
AMP_Scanner	0.76	0.81	0.70	0.81	0.51
iAMPpred	0.70	0.74	0.65	0.75	0.40
DBAASP	0.79	0.77	0.81	-*	0.57
Average	*0.79*	*0.84*	*0.75*	*0.86*	*0.59*
CalcAMP+	0.74	0.55	**0.94**	0.80	0.53
CalcAMP-	0.71	0.53	0.88	0.77	0.44

* For DBAASP, AUC-ROC cannot be calculated and ROC-curve displayed since it only returns binary results and we do not have access to the probabilities associated.

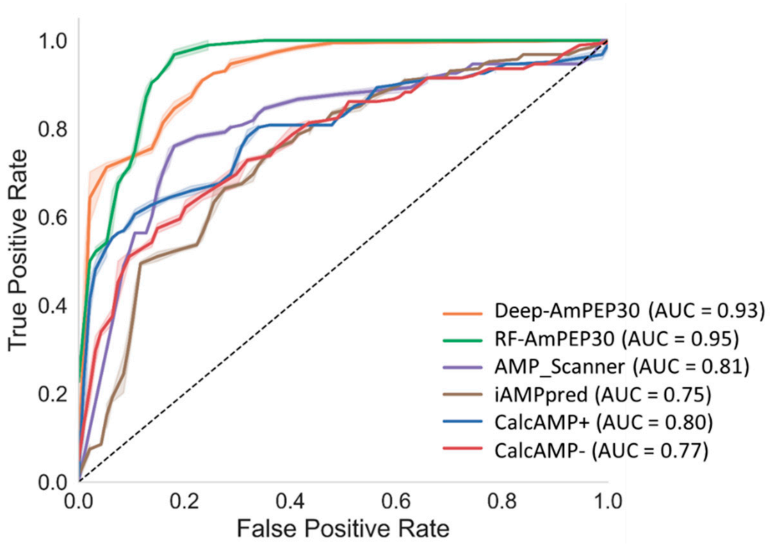

Figure S6. Receiver operator characteristic (ROC) curves of the different AMP classifiers and their area under the curve score obtained using AmPEP external benchmark dataset.

Comparison with Antimicrobial Peptide Scanner vr.2 dataset

The Antimicrobial Peptide Scanner vr.2 validation dataset is composed of 354 AMPs and 354 Non-AMPs (sequences from 11 to 172 AA in length) and was downloaded on their website: https://www.dveltri.com/ascan/v2/about.html. For this dataset, some modifications were performed, more precisely we retained only peptides with a length between 5 and 30 AA so that every prediction model are able to process it. We ended with 185 AMPs and 204 Non-AMPs. As for AmPEP, it is important to note that this dataset presents several peptides in common with the training dataset of our models. This common peptides are the same for both Gram+ and Gram- dataset and are shown in figure S7 with a confusion matrix representing how they are labelled upon datasets. In particularly, on their 185 peptides classified as AMP: 70 are common to CalcAMP training dataset whom 23 of them are labelled as Non-AMPs, contrarily to Antimicrobial Peptide Scanner vr.2 validation dataset. On their negatives peptides, none are common.

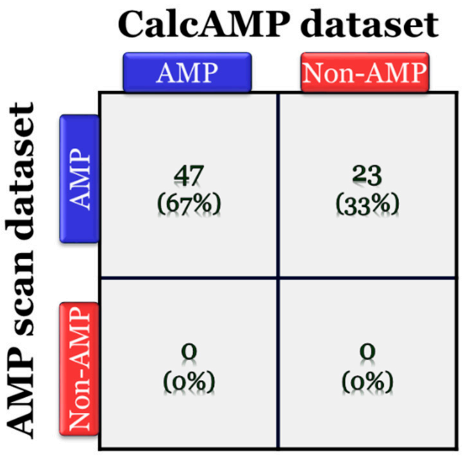

Figure S7. Matrix of labels for the common peptides between training dataset of CalcAMP+ and CalcAMP- models and adapted Antimicrobial Peptide Scanner vr.2 validation dataset.

Table S6. Comparison of different AMP prediction classifiers using adapted Antimicrobial Peptide Scanner vr.2 validation dataset.

Model	Accuracy	Sensitivity	Specificity	AUC-ROC	MCC
Deep-AmPEP30	0.88	0.91	0.85	0.93	0.77
RF-AmPEP30	0.92	0.94	0.91	0.97	0.84
AMP_Scanner	**0.96**	**0.98**	0.95	**0.99**	**0.93**
iAMPpred	0.89	0.92	0.87	0.96	0.79
DBAASP	0.85	0.82	0.88	-*	0.70
Average	*0.90*	*0.91*	*0.89*	*0.96*	*0.81*
CalcAMP+	0.77	0.52	**1.00**	0.83	0.59
CalcAMP)	0.69	0.40	0.95	0.71	0.43

* For DBAASP, AUC-ROC cannot be calculated and ROC-curve displayed since it only returns binary results and we do not have access to the probabilities associated.

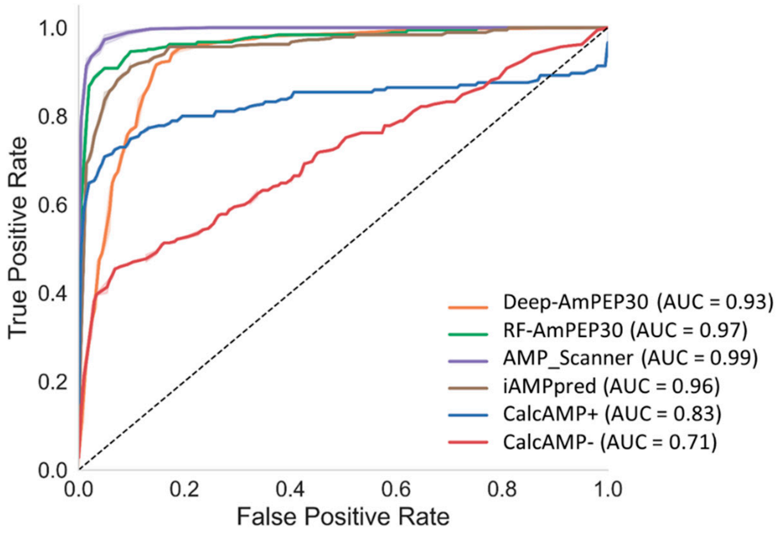

Figure S8. Receiver operator characteristic (ROC) curves of the different AMP classifiers and their area under the curve score obtained using adapted Antimicrobial Peptide Scanner vr.2 validation dataset.

- **CalcAFP prediction results**

Table S7. Results of ML classifiers created for AFP prediction. The values in brackets represent the standard deviation obtained via 10-fold cross validation.

Model	Accuracy	Sensitivity	Specificity	AUC-ROC	MCC
RF_tuned	0.78 (0.03)	0.66 (0.06)	0.86 (0.04)	0.85 (0.03)	0.54 (0.06)
RF_tuned_sel	0.78 (0.02)	0.65 (0.03)	0.87 (0.02)	0.85 (0.02)	0.54 (0.04)
ET_tuned	0.78 (0.03)	0.65 (0.06)	0.87 (0.02)	0.84 (0.03)	0.53 (0.06)
ET_tuned_sel	0.78 (0.03)	0.65 (0.05)	0.87 (0.03)	0.84 (0.03)	0.53 (0.06)

- **Comparison with other datasets**

CalcAFP was compared to three existing tools freely available:
- iAMPpred (http://cabgrid.res.in:8080/amppred/server.php)
- ClassAMP, SVM model (http://www.bicnirrh.res.in/classamp/predict.php)
- Antifp, Main_binary_model3 (https://webs.iiitd.edu.in/raghava/antifp/predict3.php)

Comparison with Antifp dataset

The Antifungal dataset 3 (Antifp_Main) validation dataset is composed of 291 AFPs and 291 Non-AFPs (sequences from 4 to 100 AA in length) and was downloaded on their website: https://webs.iiitd.edu.in/raghava/antifp/algo.php. For this dataset, some modifications were performed, more precisely only peptides with a length between 5 and 35 AA were retained so that every prediction model are able to process it. We ended with 58 AFPs and 67 Non-AFPs. Once again a few peptides of this dataset are common to the training dataset of our models. This common peptides are shown in figure S9 with a confusion matrix representing how they are labelled upon datasets. In particularly, on their 58 peptides classified as AFP: 7 are common to CalcAFP training dataset whom 2 of them are labelled as Non-AFPs, contrarily to Antifp main validation dataset. On their negatives peptides, only 2 are common whom 1 is not classified similarly.

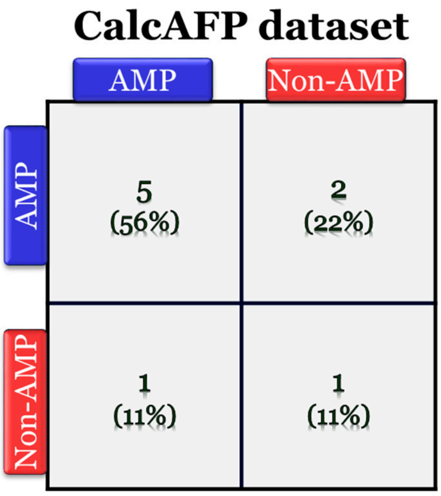

Figure S9. Matrix of labels for the common peptides between training dataset of CalcAFP model and adapted Antifp main validation dataset.

Table S8. Comparison of different AFP prediction classifiers using adapted Antifp main validation dataset.

Model	Accuracy	Sensitivity	Specificity	AUC-ROC	MCC
iAMPpred	0.61	0.59	0.63	**0.63**	0.21
ClassAMP	0.37	0.33	0.40	-*	-0.27
Antifp	**0.82**	**0.78**	**0.85**	-*	**0.63**
Average	*0.60*	*0.57*	*0.63*	-	*0.19*
CalcAFP	0.48	0.12	0.79	0.46	-0.12

* For ClassAMP and Antifp, AUC-ROC cannot be calculated and ROC-curve displayed since it only returns binary results and we do not have access to the probabilities associated.

Since the probabilities associated to the prediction from half of the tested model are not accessible, the ROC-Curve associated to these results were not displayed.

Disclaimer/Publisher's Note: The statements, opinions and data contained in all publications are solely those of the individual author(s) and contributor(s) and not of MDPI and/or the editor(s). MDPI and/or the editor(s) disclaim responsibility for any injury to people or property resulting from any ideas, methods, instructions or products referred to in the content.
